# ASO Author Reflections: Impact of Merkel Cell Polyomavirus Antibody in Tumor and Plasma Specimens

**DOI:** 10.1245/s10434-024-16381-8

**Published:** 2024-10-28

**Authors:** Ryan K. Schmocker, Laura M. Enomoto

**Affiliations:** Department of Surgery, University Surgical Oncology, University of Tennessee Graduate School of Medicine, Knoxville, TN USA

## Past

Merkel cell carcinoma (MCC) remains an aggressive and deadly neuroendocrine carcinoma of the skin. Carcinogenesis of MCC is associated with Merkel cell polyomavirus (MCPyV) infection in the majority of patients in the USA^1^. Previous work has demonstrated that the presence of MCPyV-Ab in the plasma offers important prognostic information, with patients with plasma titers positive for MCPyV-Ab experiencing a significantly longer disease-free survival (DFS) compared with those patients who were MCPyV-Ab negative^2^. However, there is a paucity of data examining the role of MCPyV-Ab in the tumor specimens in the context of the MCPyV-Ab plasma status, and whether MCPyV-Ab in the tumor offers similar prognostic information.

## Present

In the first known study investigating the impact of MCPyV-Ab in tumor tissue, we demonstrated a trend in improved 2 year DFS and overall survival (OS) in patients positive for MCPyV-Ab compared with those who were MCPyV-Ab^3^. This trend suggests that tumor MCPyV-Ab positivity may also serve as a prognostic factor similar to the presence of plasma MCPyV-Ab positivity. We also discovered two subgroups of patients with discordance between tumor and plasma MCPyV-Ab status, the implication of which is unclear.

## Future

The role of MCPyV-Ab in plasma and tumor specimens continues to expand. Our discovery of two subgroups of patients with discordant tumor and plasma MCPyV-Ab status highlights our continued lack of understanding of the cellular and immunologic factors that regulate MCPyV transcription during either viral infection or the development of MCC. Due to the rarity of the disease, a multi-institutional cohort will likely be required to attain the statistical power and control for confounding factors to draw more definitive conclusions. Our current study offers a framework on which to base further investigation, as well as impetus for continued molecular studies to elucidate the mechanisms responsible for the development of MCC.
